# Left ventricular haemodynamic effects in a patient with endocarditis

**DOI:** 10.1007/s12471-021-01615-1

**Published:** 2021-08-17

**Authors:** M. A. W. Habets, S. Bouwmeester

**Affiliations:** Department of Cardiology, Catharina Hospital Eindhoven, Eindhoven, The Netherlands

A 53-year-old man, with no medical history, presented to the emergency department with malaise and severe shortness of breath two months after a dog bite. On physical examination, the heart rate was 95 beats per minute, blood pressure was 128/48 mm Hg and a cardiac decrescendo diastolic murmur as well as a Duroziez sign were noted. Laboratory tests showed elevated infection parameters (C-reactive protein [CRP] 105 mg/l, leucocytes 22.5 10^^^9/l), liver enzymes and N‑terminal pro-brain natriuretic peptide (NT-pro-BNP: 3532 pg/ml). Echocardiography revealed a large vegetation on the aortic valve with severe regurgitation (Fig. 1a). A colour M‑mode Doppler imaging of the transmitral flow is shown (Fig. 1b). What is your diagnosis?

**Fig. 1:**
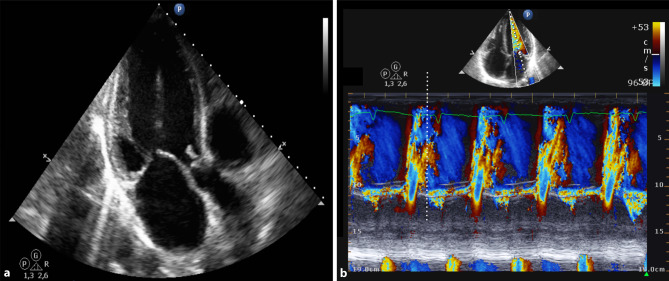
**a** Still frame from apical three-chamber view; **b** Colour M‑mode of transmitral flow from the apical four-chamber view

## Answer

You will find the answer elsewhere in this issue.

